# USP11 induce resistance to 5-Fluorouracil in Colorectal Cancer through activating autophagy by stabilizing VCP

**DOI:** 10.7150/jca.52158

**Published:** 2021-02-22

**Authors:** Hongze Sun, Rangrang Wang, Yuan Liu, Haitao Mei, Xueni Liu, Zhihai Peng

**Affiliations:** 1 Department of General Surgery, Shanghai General Hospital, Shanghai Jiao Tong University School of Medicine, Shanghai, China.; 2 Department of General Surgery, Qilu Hospital of Shandong University, Jinan, China.; 3 Department of General Surgery, Xiang'an Hospital of Xiamen University, School of Medicine, Xiamen University, Xiamen, China.

**Keywords:** USP11, VCP, colorectal cancer, 5-fluorouracil, chemotherapy resistance

## Abstract

Chemotherapy plays an important role in the treatment of patients with colorectal cancer (CRC). However, the resistance to chemotherapy severely affects the prognosis of CRC patients and the mechanisms are still poorly understood. Our study investigated the role of ubiquitin-specific protease 11 (USP11) in CRC chemotherapy and found that USP11 could induce resistance to 5-fluorouracil by activating autophagy. A series of *in vitro* and *in vivo* experiments revealed that USP11 promoted autophagy through AMPK/Akt/mTOR pathway via stabilizing valosin-containing protein (VCP). Overall, our study demonstrated that USP11 might be valuable to predict the chemotherapeutic sensitivity and improve the prognosis of CRC patients.

## Introduction

Colorectal cancer (CRC) is a common digestive tract malignant tumor and has the third cancer-related death worldwide 1. In the clinical practice, surgical operation is the most important treatment for colorectal cancer. Chemotherapy, radiotherapy (for rectal cancer), biologic agents (anti-VEGF or anti-EGFR antibody), targeted therapy and immunotherapy also play important roles in the systemic treatment of CRC patients 2. The adjuvant or neoadjuvant chemotherapy based on 5-fluorouracil (5-Fu) is recommended for stage III or stage II colorectal cancer patients with high risk factors 2. The 5-Fu mono-chemotherapy or the chemotherapy regimens combined with leucovorin, irinotecan or oxaliplatin have significantly improved the prognosis of patients with colorectal cancer 3, 4. However, due to the complexity of pathogenesis, the sensitivity to chemotherapy drugs varies greatly in different CRC patients and the drug resistance severely affects the clinical prognosis of CRC patients 5. For example, up to 40% of CRC patients receiving 5-Fu-based adjuvant chemotherapy experience recurrence or die within 8 years. Furthermore, nearly 50% of CRC patients with metastasis are resistant to chemotherapy based on 5-Fu and the 5-year survival rate is as low as 12% 6. In clinical practice, *RAS* and *RAF* (*KRAS, NRAS*, and *BRAF*) mutations are tested to predict the response of CRC patients to anti-EGFR therapy 3, 7. Therefore, we think the discovery of novel tumor markers that could predict the sensitivity of CRC patients to 5-Fu is also invaluable for the personalized treatment.

Autophagy could degrade and recycle intracellular proteins and organelles through lysosomes to keep the metabolic homeostasis 8. Autophagy plays a double-edged sword role in the development of cancer. On the one hand, autophagy could inhibit cancer by clearing away the damaged proteins and organelles that may promote tumorigenesis. On the other hand, autophagy may promote cancer by enabling tumor cells to tolerate the microenvironment, such as starvation, hypoxic and tumor-induced inflammation 9. It has been reported that autophagy may be utilized by cancer cells to induce resistance to chemotherapy drugs 10. Recent studies have found that the activation of autophagy is closely related to the 5-fluorouracil resistance in colorectal cancer 11-13. Therefore, identifying the mechanism of autophagy pathway might provide new approaches to colorectal cancer therapy.

Valosin-containing protein (VCP, also called p97) is a hexameric AAA+-type ATPase and each subunit contains two ATPase domains and one regulatory N-terminal domain. The protein could govern various cellular processes including degradation of damaged proteins and organelles, chromatin regulation and regulation of key signaling pathways 14. The mechanisms of VCP function are directed by ubiquitin and based on a series of co-factors 15. VCP could govern cellular stress responses through binding the ubiquitinated proteins and facilitating their degradation mediated by proteasome 16. Of note, VCP also has an impact on the lysosomal system and autophagy, the aberrant autophagy process is the prominent feature of disease caused by VCP dysfunction 17. In summary, VCP plays an important role in both autophagy and the ubiquitin-proteasomes pathway.

There are two main protein degradation pathways: autophagy mediates the substrate to be degraded by lysosomes, another one is ubiquitin-proteasomes pathway which is a mechanism for degradation of proteins tagged with polyubiquitin 9. Moreover, ubiquitin-specific proteases have been reported to be involved in autophagy by deubiquitination 18, 19. Ubiquitin-specific protease 11 (USP11) was reported to induce tumor resistance to treatment through stabilizing downstream proteins by deubiquitination 20. We have proved that USP11 could promote colorectal cancer through stabilizing PPP1CA in our previous study 21. However, it's still unknown whether USP11 is relevant with autophagy and affects the efficacy of chemotherapy in colorectal cancer.

In the present study, we demonstrated that USP11 could induce resistance to 5-Fu in colorectal cancer both *in vitro* and *in vivo* via activation of autophagy through stabilizing VCP. These findings contribute to improving our understanding of the molecular mechanisms of chemotherapy resistance in CRC, and these novel biomarkers may contribute to the development of a novel therapeutic strategy for CRC.

## Material and methods

### Reagents

Antibodies against GAPDH, VCP, P62, LC3B, β-Actin, AMPK, p-AMPK, Akt, p-Akt, mTOR, p-mTOR, Beclin-1 were purchased from Cell Signaling Technology (Danvers, MA, USA). Antibody against USP11 was from Abcam (Cambridge, UK). Myc-tag (Millipore, Billerica, MA, USA) and Flag-tag (Sigma-Aldrich, Munich, Germany) antibodies were used in immunoprecipitation assays. Chloroquine (CQ), 3-Methyladenine (3-MA) and 5-fluorouracil (5-Fu) were purchased from Sigma-Aldrich (Munich, Germany), oxaliplatin (OXA), MG-132 and rapamycin were bought from Selleck company (Houston, TX, USA). The psPAX2 and pMD2.G plasmids for lentiviral construction, overexpression and knockdown plasmids for USP11 and VCP were all purchased from Shanghai OBiO and Shanghai Genechem company. The DAPRed - Autophagy Detection kit was from Dojindo (Tokyo, Japan).

### Cell culture and transfection

The cell lines HCT116, HCT8 and HEK-293T cells were obtained from Chinese Academy of Sciences and maintained at 37 °C in a humidified incubator with 5% CO_2_. HCT116 cells were cultured in McCoy'5a medium supplemented with 10% FBS, HEK293T cells were cultured in DMEM culture medium supplemented with 5% FBS, and HCT-8 cells were maintained in RPMI-1640 culture medium supplemented with 10% FBS. The chemotherapy-resistant CRC cell lines were established in our laboratory by treating HCT116 or HCT8 cells with gradually increasing concentration of 5-Fu or OXA. Cell transfection was conducted by using Lipofectamine 2000 (Invitrogen, Carlsbad, CA, USA) according to the manufacturer's protocol. The construction of stable cell line was described in our previous study 21.

### Cell viability and colony formation assays

Cell viability and proliferation were assessed using a CCK-8 kit (Dojindo, Tokyo, Japan). Cells were seeded per well in 96-well plates and treated with different reagents. The absorbance was determined using a spectrophotometer at 450 nm at the end of CCK8 incubation time. For colony formation, 1000 cells per well were seeded in six-well plates and treated with the reagents. After 2 weeks, cell clones were fixed and stained using crystal violet.

### Western blot

Total protein from cells was extracted with RIPA lysis buffer and the protein concentrations were quantified with BCA protein assay kit (Pierce, Rockford, IL, USA). Equal amounts of protein were separated using SDS-PAGE and transferred to PVDF membranes. The membranes were incubated with primary antibody at 4 °C overnight after being blocked and antibodies against GAPDH or β-Actin were used as loading controls. Horseradish peroxidase-conjugated secondary antibodies were used and the protein signals were visualized using electrochemiluminescence detection reagents.

### Autophagosomes and autolysosomes detection

DAPRed - Autophagy Detection kit was used to detect the formation of autophagosomes and autolysosomes. Wash the adhered cells with culture medium and add appropriate volume of 0.2 μM DAPRed working solution. Incubate the cells at 37 °C for 30 minutes and remove the supernatant. Treat the cells with culture medium contained 5-Fu (80 μM or 100 μM) and incubate at 37 °C for 18 hours. The autophagosomes and autolysosomes were detected by a confocal fluorescence microscope.

### Co-immunoprecipitation (Co-IP)

Total proteins were prepared by IP lysis buffer (Pierce, Rockford, IL, USA) containing protease inhibitors. For interaction between USP11 and VCP, cell lysates (300 μg) were incubated with 1-2 μg antibody at 4 °C overnight. Then, protein A/G magnetic beads were added into the cell lysates and incubated at 4 °C for 1 hour. Precipitates were washed with cold lysis buffer before detected by immunoblotting.

### Cycloheximide and MG-132 treatment assays

Cells were treated with 100 μg/mL cycloheximide (MedChemExpress, Monmouth Junction, NJ, USA) and/or 15 μM MG-132 (Selleck, Houston, TX, USA) for the indicated times, followed by Western blotting.

### Subcutaneous xenograft assays

Male nude mice, 4-week-old, were purchased from Shanghai SLAC Laboratory Animal Company. Animal experiments were approved by the Institutional Animal Care and Use Committee of the Shanghai Jiao Tong University School of Medicine. 1 × 10^6^ cells were injected subcutaneously into the right flanks of nude mice (three mice per group). Seven days after the inoculation of tumor cells, each mouse was treated with 5-Fu 100 mg/kg combined with CQ 60 mg/kg or not through intraperitoneal injection twice a week. Tumor size was measured using Vernier calipers and the volume was calculated using the formula: volume = length × width^2^ × 0.5. All mice were euthanized after 3 weeks treatment, and tumors were dissected and fixed in formalin.

### Statistics

Quantitative variables were analyzed by Student's *t* test between groups. One-way ANOVA was used for multiple group comparisons. All the data were processed by SPSS 22.0 (IBM Statistics, Armonk, NY, USA) and GraphPad Prism 7 (San Diego, CA, USA). *P* < 0.05 was considered statistically significant.

## Results

### USP11 is associated with the sensitivity of CRC cells to 5-Fu

Chemotherapy resistance remains the most common and major cause of clinical chemotherapy failure and leads to poor prognosis among CRC patients. We have shown that USP11 could promote colorectal cancer, then we wondered whether USP11 is involved in chemotherapy resistance in CRC patients. In clinical practice, 5-Fu and oxaliplatin are commonly used in the adjuvant or neoadjuvant chemotherapy among CRC patients, so we investigated the sensitivity of CRC cells with different USP11expression levels to these two drugs.

**Figure 1:**
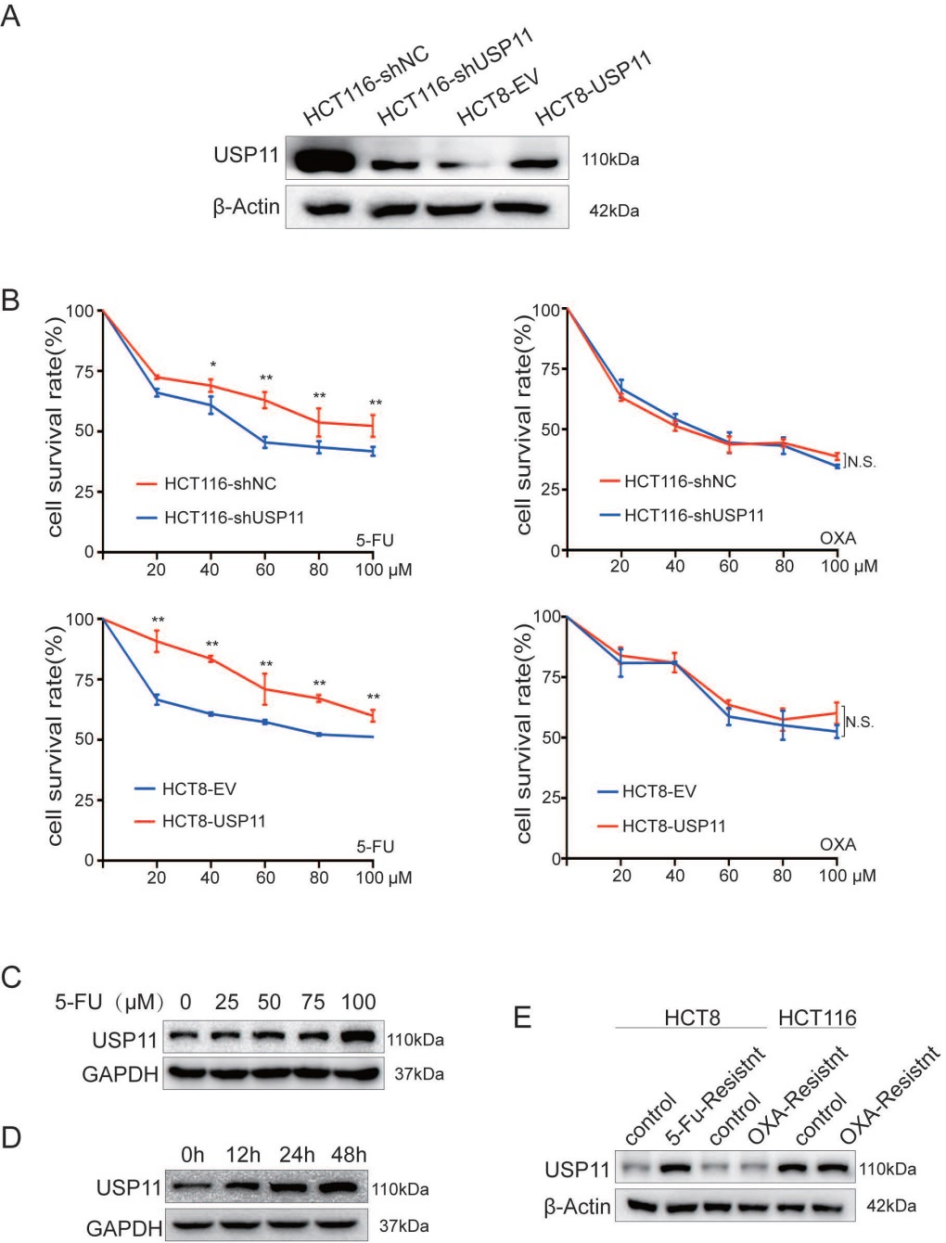
** USP11 is associated with the sensitivity of CRC cells to 5-Fu. A.** Western blot analysis of USP11 expression in stable cell lines. **B.** The sensitivity of different stable cell lines to 5-fluorouracil and oxaliplatin was determined by CCK-8 assay. **C.** The effect of 5-fluorouracil concentration on the expression level of USP11 in HCT8 cells was determined by Western Blot. **D.** The relationship between the treatment time of 5-fluorouracil and the expression level of USP11 in HCT8 cells was determined by Western Blot. E. USP11 expression levels in chemotherapy-resistant CRC cells. Error bars indicate the standard deviation of triplicates. *P < 0.05, **P < 0.01, N.S., no significant difference, Student's *t* test.

Firstly, we constructed HCT116-shUSP11/NC, HCT8-USP11/EV cell lines with different USP11 expression levels (Figure 1A). Next, we treated the cells with different concentrations of 5-Fu and oxaliplatin, and then the relative cell viability was measured by CCK-8 assay. The results showed that USP11 overexpression significantly enhanced the resistance of HCT8 cells to 5-Fu compared with the control group, whereas the opposite effect was observed in USP11 knockdown HCT116 cells (*P* < 0.05, Student's *t* test). However, the expression level of USP11 did not affect the sensitivity of cells to oxaliplatin (Figure 1B). Then, we selected HCT8 cells which has low expression level of USP11 for subsequent studies. After 48 hours of treatment with different concentrations of 5-Fu, we found that 5-Fu dramatically upregulated USP11 expression in a dose-dependent manner in HCT8 cells (Figure 1C). We further treated HCT8 cells with 100 μM 5-Fu for different time and found that the expression level of USP11 was also positively correlated with the time of 5-Fu treatment (Figure 1D). Furthermore, we detected USP11 expression in chemotherapy-resistant CRC cell lines which were constructed in our laboratory previously, and USP11 overexpression was only found in 5-Fu-resistant CRC cells but not OXA-resistant CRC cells (Figure 1E). The above results indicated that the expression level of USP11 might be relevant with resistance to 5-Fu chemotherapy in CRC cells.

### USP11 is related to the activation of autophagy in colorectal cancer cells

**Figure 2:**
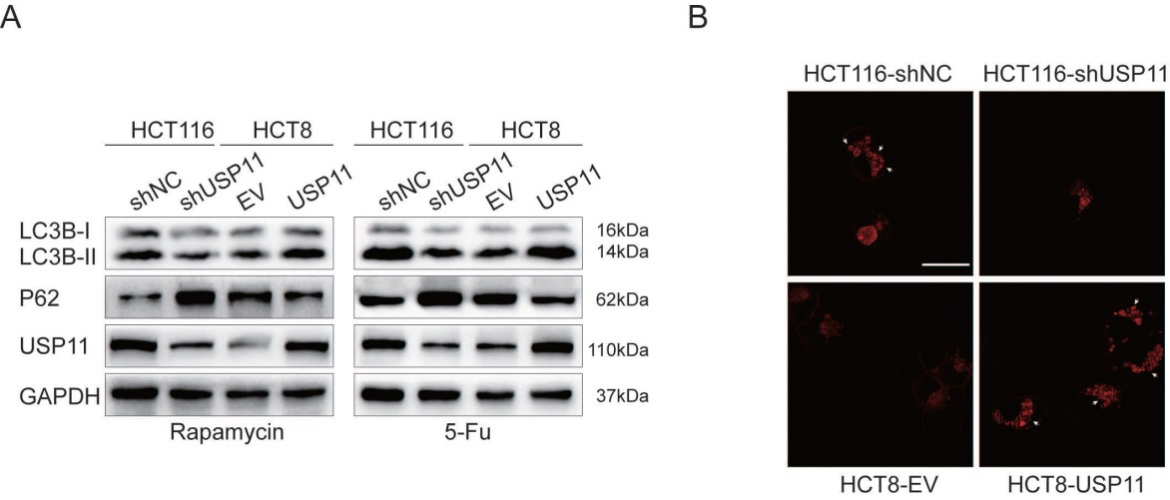
** USP11 is related to the activation of autophagy in CRC cells. A.** Autophagy related proteins were detected after the cells were treated by rapamycin (500 nM) and 5-fluorouracil (80 μM in HCT116 and 100 μM in HCT8) respectively. **B.** The formation of autophagosomes and autolysosomes was detected by DAPRed (white arrows); Scale bar 25 μm.

It has been reported that the activation of autophagy could induce resistance to chemotherapy drugs in tumor cells 11. To investigate whether USP11 is related to autophagy, rapamycin and 5-Fu were used to stimulate USP11 knockdown and overexpressing cells. We found that USP11 knockdown significantly increased the expression of autophagy marker P62 and the LC3B-II/I ratio was reduced compared with the control group, while opposite trend was observed in HCT8-USP11 cells compared with HCT8-EV cells (Figure 2A).

In the process of autophagy, the damaged proteins or organelles can form autophagosomes and autolysosomes composed of double membrane. The detection of autophagosomes and autolysosomes was commonly used as a measurement for autophagy. Since the stable cell lines we constructed were labeled with GFP, we used DAPRed as a novel method to detect autophagy 22. The autophagosomes and autolysosomes were observed by confocal microscopy after the cells were treated with 5-Fu. The results showed that the number of autophagosomes and autolysosomes formed in HCT116-shNC cells was significantly higher than that in HCT116-shUSP11 cells, while reverse trend was observed in HCT8-USP11 cells compared with HCT8-EV cells (Figure 2B). Taken together these data, we hypothesized that USP11 might mediate resistance to 5-Fu by inducing autophagy in colorectal cancer cells and verified the hypothesis in follow-up experiments.

### USP11 might interact with and stabilize VCP

To explore the underlying mechanism of autophagy activation induced by USP11, we analyzed the differential proteins identified in our previous study and found that VCP (valosin-containing protein), which has been proved to be involved in autophagy might play an important role in this process 23.

**Figure 3:**
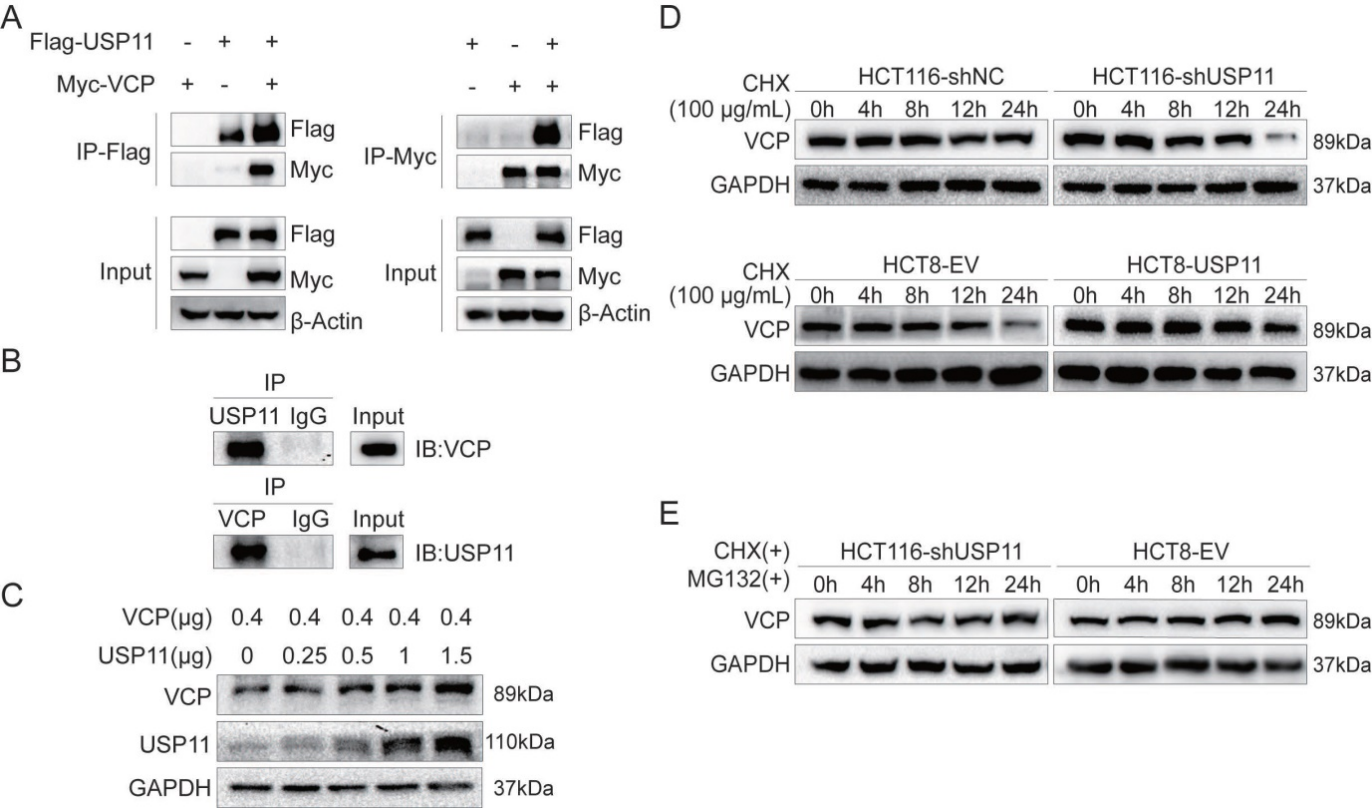
** USP11 interacts with and stabilizes VCP. A.** Exogenous USP11/VCP interaction in HEK293T cells transfected with Flag-USP11 and Myc-VCP plasmids was confirmed by Co-IP assays. **B.** Endogenous formation of the USP11/VCP complex in HCT116 cells was validated by Co-IP assay. IgG antibodies were used as controls. **C.** VCP plasmid (0.4 μg) and the indicated doses of USP11 plasmid (0, 0.25, 0.5, 1 and 1.5 μg) were co-transfected into HEK293T cells. Cell protein was harvested 48 hours after transfection and analyzed by Western blot. **D.** Effects of USP11 on the degradation of VCP. HCT116-shNC/shUSP11 cells and HCT8-EV/USP11 cells were exposed to CHX (100 µg/mL) and harvested at the indicated times. VCP was measured using Western blot. **E.** Degradation of VCP was alleviated when cells were co-treated with CHX and MG-132 (15 µM).

To test our hypothesis, we performed exogenous reciprocal Co-IP assays by co-transfecting Flag-USP11 and Myc-VCP into HEK293T cells. The results showed that Myc-VCP was precipitated with Flag-USP11 using an anti-Flag antibody. Similarly, Flag-USP11 was also precipitated using an anti-Myc antibody (Figure 3A). HCT116 cells were lysed for endogenous Co-IP assay by using anti-USP11 or anti-VCP antibody, and the precipitated proteins were subjected to western blot using anti-VCP or anti- USP11 antibody as indicated. The results revealed that USP11 can form a complex with VCP in HCT116 cells (Figure 3B). These findings confirmed the interaction between USP11 and VCP.

As a deubiquitinating enzyme, USP11 predominantly stabilizes downstream proteins via post-translational modification. HEK293T cells were transfected with increased amount of USP11 and equal amount of VCP plasmids. We found that USP11 upregulated VCP expression in a dose-dependent manner (Figure 3C). Furthermore, cycloheximide (CHX, 100 μg/mL) was used as protein synthesis inhibitor to analyze the half-life of VCP. The results showed that knockdown or overexpression of USP11 resulted in decreased or increased protein stability of VCP respectively compared with the controls (Figure 3D). We also found that the VCP degradation caused by low USP11 expression was rescued by proteasome inhibitor MG-132 (Figure 3E). These findings suggested that USP11 could interact with VCP and prevent its degradation.

### USP11 promotes autophagy through AMPK/Akt/mTOR signaling pathway in a VCP-dependent manner

**Figure 4:**
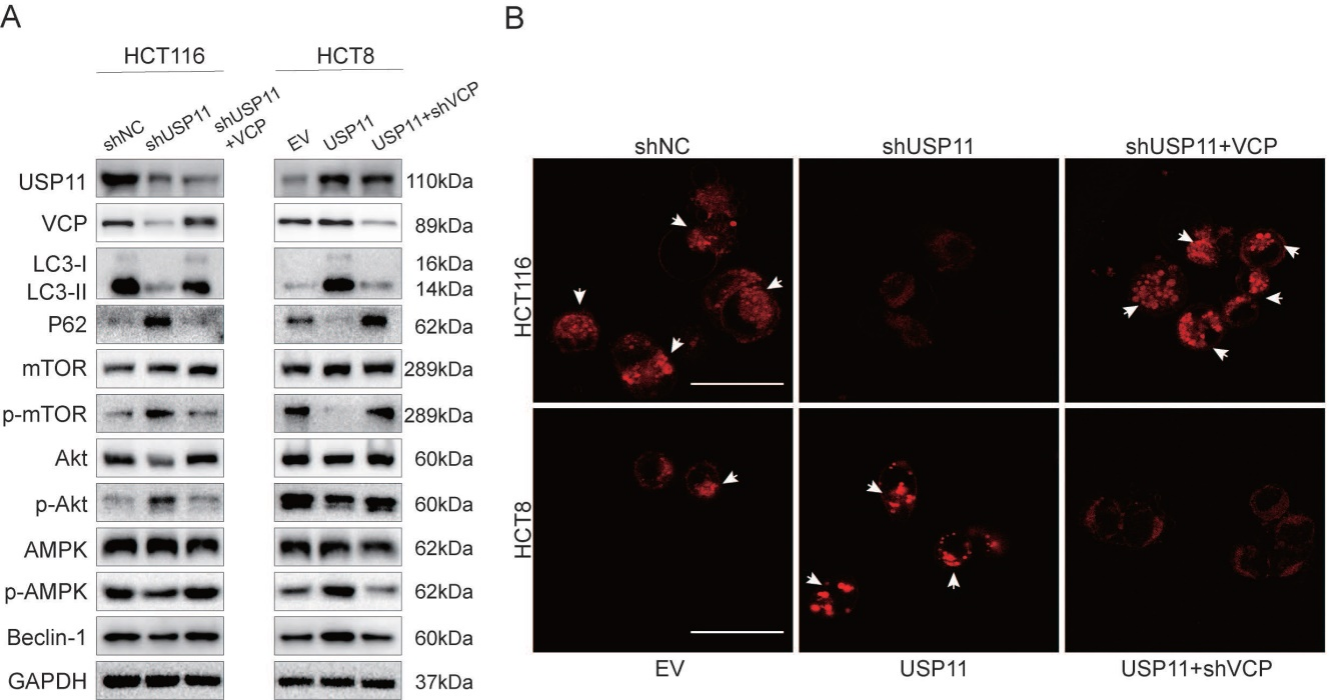
** USP11 promotes autophagy through AMPK/Akt/mTOR signaling pathway via VCP-dependent manner. A.** Involvement of VCP on USP11-mediated activation of the AMPK/Akt/mTOR pathway. **B.** The effect of VCP on the USP11-induced formation of autophagosomes and autolysosomes (white arrows); scale bar 50 μm.

Our previous experiments have confirmed that USP11 can promote autophagy in colorectal cancer cells, however, the underlying mechanisms were still unknown. It has been reported that PI3K/Akt/mTOR pathway and AMPK pathway are the most common pathways regulating autophagy activity 24, 25. Therefore, in order to investigate the relevant mechanism, we detected the key proteins involved in these pathways by Western Blot. After being treated with 5-Fu, knockdown of USP11 in HCT116 cells increased the phosphorylation levels of Akt and mTOR but decreased the phosphorylation level of AMPK. Interestingly, VCP overexpression could partially rescue the phosphorylation level changes caused by USP11 knockdown. What's more, the opposite trend was observed in HCT8 cells after overexpression of USP11. Similarly, these changes could also be rescued by VCP knockdown (Figure 4A).

We also detected the formation of autophagosomes and autolysosomes using DAPRed. The results showed that the number of autophagosomes and autolysosomes in HCT116-shUSP11 cells was lower than HCT116-shNC, and VCP overexpression could revert this change partially. Opposite trend could be observed in HCT8-USP11 cells compared with HCT8-EV cells and VCP knockdown also rescued this change (Figure 4B). Collectively, these findings suggest that USP11 could promote autophagy via AMPK/Akt/mTOR signaling pathway dependent on VCP.

### USP11 promotes 5-Fu resistance through inducing autophagy dependent on VCP

The above experimental results have confirmed that USP11 could not only mediate the resistance to 5-Fu, but also induce autophagy in colorectal cancer cells. What's more, 5-Fu treatment could increase the expression level of USP11 but had no effect on VCP (Figure 1C, D and S1). It has been reported that autophagy could induce cancer cells to chemotherapy resistance 10. Thus, we hypothesized that USP11 could promote resistance to 5-Fu in colorectal cancer by inducing autophagy. In order to verify our hypothesis, we conducted *in vivo* and *in vitro* experiments respectively.

**Figure 5:**
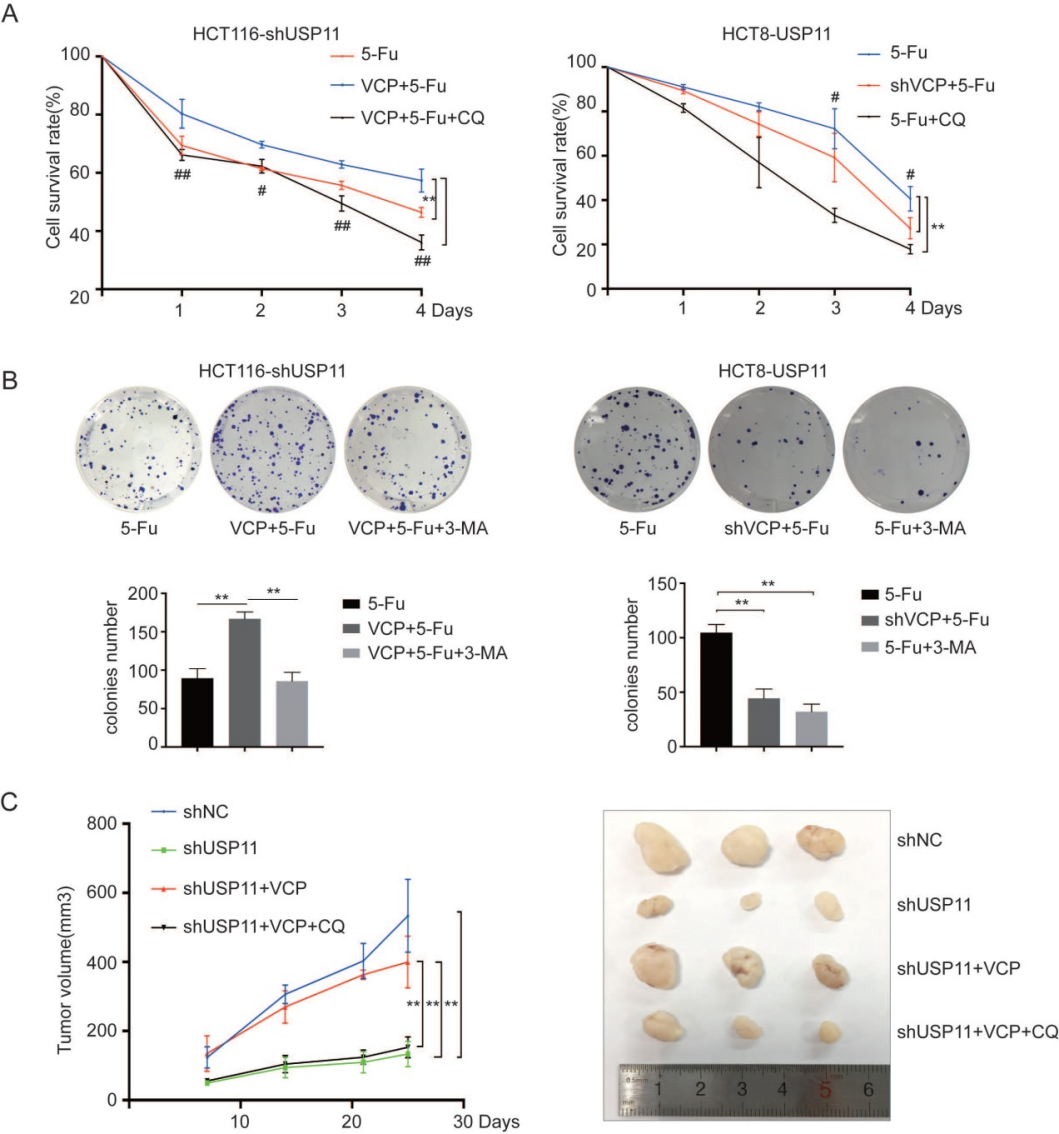
** USP11 promotes 5-Fu resistance by inducing autophagy dependent on VCP. A** and **B.** The effects of VCP on USP11-induced cell viability were investigated using CCK-8 assays and colony formation assays, CQ (20 μM) and 3-MA (10 mM) were added as autophagy inhibitors. **C.** Effects of VCP and autophagy inhibitor on the growth rate of subcutaneous tumor caused by USP11 knockdown in HCT116 (n=3). Error bars indicate the standard deviation of triplicates. *P < 0.05, **P < 0.01, *P* values were calculated with one-way ANOVA in A, C and Student's *t*-test in B. In A, #* P* < 0.05, ## *P* < 0.01 were also used to distinguish the comparison between different groups.

First, HCT116-shUSP11, HCT116-shUSP11-VCP and HCT8-USP11, HCT8-USP11-shVCP cells were treated with 5-Fu alone or 5-Fu combined with autophagy inhibitor chloroquine (CQ), and then cell viability was measured using CCK-8 assays. The results showed that overexpression of VCP inhibited reduced cell viability caused by USP11 knockdown when treated with 5-Fu. However, this phenomenon could be significantly inhibited when combine treated with 5-Fu and CQ. Similar results were also seen when knockdown VCP in HCT8-USP11 cells. In addition, we have also seen the use of autophagy inhibitor CQ significantly increases the sensitivity of HCT8-USP11 cells to 5-Fu (Figure 5A). The colony formation assays in which 3-MA was used as autophagy inhibitor also coincided with our findings (Figure 5B). What's more, inhibitors of VCP and AMPK/Akt/mTOR pathway had no influence on the proliferation of above CRC cells when treated with 5-Fu simultaneously (Figure S2).

For further investigation, we used HCT116-shUSP11, HCT116-shNC and HCT116-shUSP11-VCP cells to establish subcutaneous xenograft model in nude mice. One week later, the nude mice were treated with 5-Fu 100mg/kg combined with CQ 60mg/kg or not through intraperitoneal injection twice a week. We found that overexpression of VCP could rescue tumor growth inhibition caused by USP11 knockdown. Furthermore, CQ could inhibit tumor growth saved by VCP overexpression (Figure 5C). In conclusion, these findings confirm that USP11 could promote colorectal cancer cells resistance to 5-Fu through inducing autophagy dependent on VCP.

## Discussion

The development of colorectal cancer is a complex process involving multiple biological processes, study of related mechanisms will provide new evidence for precise treatment of CRC. In clinical practice, adjuvant or neoadjuvant chemotherapy is important treatment approach for patients with colorectal cancer. 5-fluorouracil and oxaliplatin are the most commonly used drugs in chemotherapeutic regimens, but the sensitivity to chemotherapeutics varies widely and resistance to chemotherapeutics often leads to chemotherapy failure 26, 27. Thus, the discovery of novel biomarkers to predict chemosensitivity is necessary to improve the prognosis of CRC patients.

USP11 is involved in the development and progression of multiple tumors 28-30, and we have found that USP11 could promote CRC by stabilizing PPP1CA through deubiquitination 21. However, the function and molecular mechanism of USP11 in the CRC chemotherapy are still poorly known. In the present study, we respectively used 5-Fu and oxaliplatin to treat the stable cell lines with different expression levels of USP11. The experimental results showed that USP11 overexpression could enhance CRC cells resistance to 5-Fu, and knockdown of USP11 could result in CRC cells being more vulnerable to 5-Fu. This indicates that USP11 could play important roles in the resistance to 5-Fu of colorectal cancer cells.

The research focused on 5-Fu resistance mechanism has found that some micro- RNAs, P38/MAPK pathway inactivation and Bcl protein family can participate in this process 11, 31, 32. Recently, the activation of cell autophagy process is reported to be closely related to 5-Fu resistance in colorectal cancer 12, 13. USP8, USP30 and USP35, members of ubiquitin specific protease family, could participate in the autophagy via deubiquitination 18, 19. What's more, the Spautin-1 inhibitor could inhibit autophagy by reducing the activity of USP10 and USP13 33. In this study, we found that the autophagy of colorectal cancer cells with overexpression of USP11 was significantly enhanced after treated with 5-Fu, while the autophagy of colorectal cancer cells with knockdown of USP11 was significantly weakened after treated with 5-Fu.

Studies have shown that autophagy plays a double-edged sword role in tumor progression by inhibiting or promoting cancer. Autophagy inhibits tumor progression by maintaining genomic stability, clearing endogenous reactive oxygen species, removing oncogenic proteins and inducing immune response 34. However, autophagy can also promote tumor progression by providing necessary nutrients mediating tumor cells adapt to hypoxic tumor microenvironments and inducing tumor angiogenesis and metastasis 35. In addition, autophagy can also induce tumor cells to develop resistance to chemotherapeutics and inhibiting autophagy can improve the anti-tumor effect of 5-Fu 36-38. The small molecular inhibitors targeted on the autophagy pathway have been proved to be effective in cancer treatment and some have been applied in clinical 10, 39. In our study, we found that USP11 could induce resistance to chemotherapy by activating autophagy. Therefore, studying the mechanism of autophagy related chemotherapeutics resistance of USP11 will help us find more effective treatment methods.

Mechanically, we identified VCP as the potential interaction protein from our LC-MS/MS results. VCP (also called p97) is an ATP-driven chaperone-like protein which has been proved to regulate ubiquitin-dependent protein degradation by proteasome 40. Recent studies have uncovered that VCP could mediate the ubiquitin-labelled substrates to be degraded by autophagy 41. The endogenous and exogenous Co-IP assays confirmed the interaction between USP11 and VCP in our research. Furthermore, the present study revealed that USP11 could promote AMPK/Akt/mTOR pathway through stabilizing VCP expression in CRC.

Mammalian rapamycin target protein (mTOR) pathway plays a major role in the regulation of autophagy 42, which can be regulated by the upstream PI3K-Akt pathway. The activated Akt could improve the activity of mTOR and further inhibit autophagy. In addition, Ras protein plays an opposing regulatory role in autophagy: it can inhibit autophagy by activating PI3K-Akt-mTOR pathway and induce autophagy through Raf-MEK1/2-ERK1/2 pathway 43, 44. Additionally, our previous study has proved that USP11 is relevant with the activation of ERK/MAPK pathway 21. The AMP-activated protein kinase (AMPK) pathway is also a common regulatory pathway of autophagy. When the intracellular energy is deficient and the ratio of ATP/AMP is reduced, AMPK can be phosphorylated and activated by LKB1, thereby inhibiting mTOR activity and activating autophagy 25. In our study, the resistance to 5-Fu induced by USP11 can be weakened by VCP knockdown or autophagy inhibitors. The findings might provide useful evidence regarding the autophagy inhibitors in CRC treatment and improve the prognosis of CRC patients.

For the colorectal cancer patients who plan to undergo neoadjuvant or adjuvant chemotherapy, it is worthful to assess the sensitivity of patients to chemotherapeutics before the execution of treatment. Individualized chemotherapy regimen can not only reduce the financial burden of patients, but also avoid delaying the optimal treatment time of CRC patients. Therefore, we hope that our findings in this study can provide theoretical basis for the treatment of colorectal cancer patients.

## Supplementary Material

Supplementary figures.

## Abbreviations

CRC: colorectal cancer
5-Fu: 5-fluorouracil
USP11: ubiquitin-specific protease 11
CQ: chloroquine
3-MA: 3-Methyladenine
OXA: oxaliplatin
FBS: fetal bovine serum
VCP: valosin-containing protein
CHX: cycloheximide
NC: negative control
EV: empty vector
