# Supplementary figures and images for: USP11 induce resistance to 5-Fluorouracil in Colorectal Cancer through activating autophagy by stabilizing VCP
Source: J Cancer. 2021 Feb 22;12(8):2317–25. doi: 10.7150/jca.52158 (PMC7974880; doi:10.7150/jca.52158)

Fig.S1

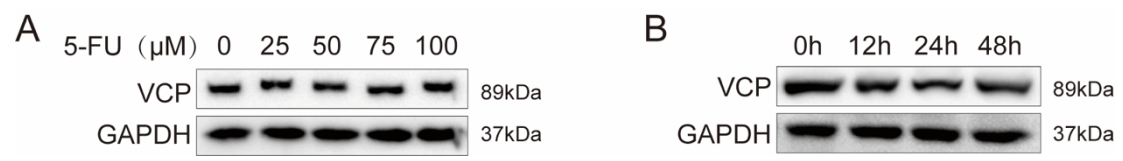

Fig.S2

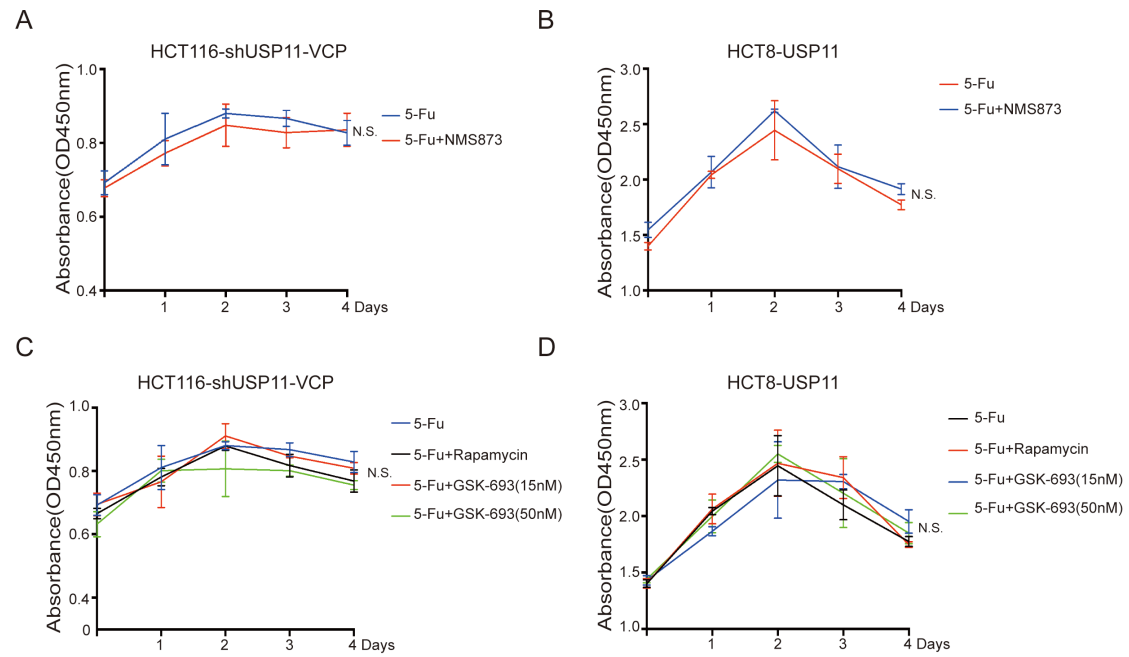

Supplement: Supplementary file 1 — Supplementary figures. [file jcav12p2317s1.pdf]
